# In Situ Characterization Reveals an Impaired Fibril Response to Loading Following Unloading during Early Achilles Tendon Healing

**DOI:** 10.1021/acsbiomaterials.5c01976

**Published:** 2026-03-28

**Authors:** Isabella Silva Barreto, Kunal Sharma, Maria Pierantoni, Md Abdul Alim, Ana Diaz, Pernilla Eliasson, Hanna Isaksson

**Affiliations:** † Department of Biomedical Engineering, 5193 Lund University, SE-223 63 Lund, Sweden; ‡ Department of Biomedical and Clinical Sciences, 4566 Linköping University, SE-581 83 Linköping, Sweden; § PSI Center for Photon Science, 28498 Paul Scherrer Institute, CH-5232 Villigen PSI, Switzerland; ∥ Department of Orthopaedics, Sahlgrenska University Hospital, SE-413 46 Gothenburg, Sweden; ⊥ Department of Orthopaedics, Sahlgrenska Academy, Gothenburg University, SE-431 80 Mölndal, Sweden

**Keywords:** nanoscale, collagen, mechanics, small-angle X-ray scattering

## Abstract

The Achilles tendon is the most frequently injured tendon in humans. Despite extensive research to understand its healing, there are still no clear rehabilitation guidelines to ensure full recovery and prevent reruptures. This could partly be due to limited understanding of how loading during tendon healing affects the multiscale mechanical response and in particular the nanoscale. We combined synchrotron small-angle X-ray scattering mapping with *in situ* tensile stress relaxation of early healing (1-, 2-, and 3 weeks) rat Achilles tendons subjected to either full activity or immobilization. Initially, *in vivo* unloading resulted in a similar collagen fibril structure and spatial distribution, but then, as healing progressed, the unloaded group exhibited more alterations in tissue distribution. While no clear differences were observed at the tissue scale, unloading clearly altered the nanoscale mechanical response already after 1 week of healing. Unloading led to an impaired nanoscale response, characterized by higher spatial variations, less fibril recruitment, and capacity for elongation. This indicates that the nanoscale of healing tendons is more susceptible to changes in the loading environment compared to the tissue scale. These insights contribute to a better understanding of the effects of *in vivo* loading on multiple length scales of the healing Achilles tendon.

## Graphical abstract

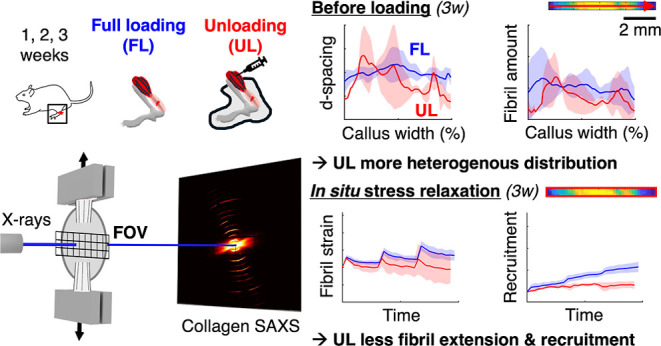

## 1 Introduction

The Achilles tendon connects the calf muscles to the heel bone and is the most frequently injured tendon in humans.^1,2^ Despite extensive research to understand the processes of tendon healing, there are still no clear rehabilitation guidelines that ensure full recovery and ruptured tendons rarely regain prerupture structural or mechanical properties.^3,4^ Additionally, the rerupture risk for both the injured and the contralateral uninjured tendon is higher than for uninjured subjects.^5^ Thus, there is a need for a better understanding of the healing process to improve rehabilitation.

During tendon healing, the tissue is formed during three overlapping phases; (1) inflammatory, (2) reparative, and (3) remodeling phase.^4,6,7^ Most studies characterizing the healing process of Achilles tendons have been conducted on full midtendon-transections in animals, specifically rodents. For these animals, the inflammatory phase was shown to last for a few days. As the tendon enters the reparative phase, intense matrix production occurs. An initial disorganized network of collagen type III is laid down, with its gene expression and quantity peaking around 1 week after healing. As the tendon enters the remodeling phase during 3 to 12 weeks after healing, this is gradually replaced by more aligned collagen type I.^8,9^ Then, the general matrix synthesis decreases and the tissue starts to transform into a more fibrous and scar-like tissue with increased interfiber bonding.^6,7^

Tenocytes (tendon fibroblast cells) embedded in the tendon matrix are mechanosensitive and respond to altered levels of physical load by modifying extracellular matrix (ECM) production and cell signaling. Thus, mechanical stimuli during the tendon healing process influence regeneration and remodeling of the collagen structure, as well as mechanical properties.^4,6^ Both insufficient and excessive loading have detrimental effects on the healing outcome.^4,10,11^ In rat Achilles tendons, reduced loading by Botox injections, initially results in smaller, shorter, and weaker tendons with reduced creep behavior, altered stiffness, and matrix gene expression.^12–15^ However, these effects seem to diminish after 4 weeks of healing.^15^ By further minimizing mechanical stimuli, e.g., by combining Botox injections with tail suspension or cast immobilization, mechanical properties and gene expression can be more severely impaired,^13,14^ resulting in a less organized matrix characterized by increased angiogenesis and fewer, less aligned, rounded cells.^8^ While these studies indicate that loading is favorable for regaining tissue integrity, high levels of loading have also been shown to increase and prolong the inflammatory response, with uncertain clinical implications.^11,16,17^ Additionally, excessive levels of loading can result in microdamage^14,16^ and scar-tissue formation.^18^ Thus, the optimal timing and loading magnitude during healing are still being debated. Furthermore, little is known regarding the effects of loading on structural regeneration at both the micro- and nanoscale. As mechanical properties of tendons are highly dependent on their multiscale structure, further investigation into this mechanostructural relationship at all length scales is crucial.

It has been hypothesized that the multiscale hierarchical structure of tendons contributes to several strain partitioning mechanisms, such as sliding of nanoscale fibrils, microscale fibers, and mesoscale subtendons or fascicles, together with shearing of the interstitial matrix between them.^19–25^ While the mechanics of tendons have been extensively studied, knowledge regarding the contribution of structures at each length scale and how they relate to each other in immature healing tendon tissue is still limited. Due to the highly periodic quarter stagger of collagen molecules into fibrils at a periodic distance (*d*-spacing) of approximately 67 nm,^26,27^ collagen fibrils can be probed using small-angle X-ray scattering (SAXS). This technique has been extensively applied to study the nanoscale structure and mechanics of intact tendons,^28^ particularly in rat tail tendons,^20,29–31^ and has been a crucial tool in obtaining our current understanding of fibril deformation mechanisms. The combination of SAXS with *in situ* loading has shown that fibrils in intact tendons respond through a combination of collagen molecule elongation, gap region extension, and relative sliding of adjacent molecules.^19,20,28–36^ Additionally, strain partitioning has been suggested, with fibril strains being substantially lower than the applied tissue strain^19,21,29–33,36^ as well as the failure strain of individual dissected fibrils,^37–39^ and with fibrils sometimes exhibiting 10–20% slower relaxation than the full tissue.^21,36^

While the application of SAXS to study the ongoing formation of collagen fibrils during healing has been limited, our previous findings showed that collagen fibril formation during rat Achilles tendon healing exhibits high spatial specificity, with preferential initial formation close to the stumps and toward one or both peripheral regions.^15,40^ Unloading during this process resulted in a more disorganized ECM with less collagenous material and delayed callus maturation as well as stump remodeling.^40^ However, how this highly specific and load-dependent tissue formation relating to the nanoscale mechanical response of the newly formed collagen fibrils remains unexplored. Using *in situ* SAXS and phase-contrast microtomography, we previously observed that unloading of intact, uninjured Achilles tendons impairs the nano- and microscale mechanical response by structural disorganization and potential alteration in strain partitioning between length scales.^21^ Additionally, our recent study using phase-contrast microtomography demonstrated a nonuniform and anisotropic response with spatial variations in 3D^41^, further highlighting the need for spatially resolved mechanical studies also at the nanoscale.

The aim of this study was to characterize the effect of different *in vivo* load levels on the collagen nanoscale mechanical response, specifically how this response varies across different regions of the healing tissue during early rat Achilles tendon healing (1-, 2-, and 3 weeks postinjury), and how it relates to the tissue-scale mechanical response. This was achieved through a combination of 2D SAXS mapping and concurrent *in situ* tensile loading.

## 2 Methods

### 2.1 Animal Experiment

Twenty-four female specific pathogen free Sprague–Dawley rats aged 10–13 weeks (Janvier, Le Genest-Saint-Isle, France) were used. The rats were housed in pairs under controlled conditions (22 °C, 55% humidity, 12 h light/dark cycle) with food and water ad libitum. They were randomly assigned into two groups (Figure 1A): (1) full loading (FL) through free cage activity and (2) unloading (UL) by Botox injections in combination with joint fixation using a steel orthosis.^14^ The Botox (Botulinum toxin, Allergan, Irvine, CA) injections were given 4 days prior to tendon transection to achieve full muscle paralysis. The injections were given in the right calf muscles (gastrocnemius medialis, gastrocnemius lateralis, and soleus) under isoflurane sedation at a total dose of 3 U (1 U per muscle). All rats underwent full transection of the right Achilles tendon under isoflurane anesthesia together with removal of the plantaris tendon. Preoperative treatment with antibiotics (engemycin, 25 mg/kg) and analgesics (temgesic, 0.045 mg/kg) were administered, with continued analgesia for 48 h postsurgery. Following Achilles tendon transection, animals in the UL group were fitted with a steel orthosis, placing the ankle in a neutral position. After 1-, 2- or 3 weeks post-transection, the rats were anesthetized and euthanized with carbon dioxide and the Achilles tendons were harvested as previously described,^14,15^ resulting in *n* = 4 per loading group and time point (Supporting Information, Table S1). The Achilles tendons were dissected together with the calcaneal bone and the gastrocnemius soleus muscle complex (calf muscle) and stored frozen in phosphate-buffered solution (PBS) until measurements. The experiment is reported according to the ARRIVE guidelines, adhered to institutional guidelines for care and treatment of laboratory animals, and was approved by the Regional Ethics Committee for animal experiments in Linköping, Sweden (ID1424).

### 2.22.5 Small-Angle X-ray Scattering and *In Situ* Loading

#### 2.2.1 Experimental Setup

SAXS measurements were carried out at the coherent SAXS beamline (cSAXS) at the Swiss Light Source (SLS), Paul Scherrer Institute (PSI), Switzerland. The cross-sectional area of the healing tendon was calculated by assuming an elliptical geometry and using the transverse and sagittal diameters measured with a digital slide caliper in the middle of the callus. Kapton film was placed around the tendons and sealed with a drop of PBS. The tendons were placed in a custom-built loading device (111N load cell, accuracy ±1%, LC201 25, Omega Engineering Inc. US),^42,43^ as described in our earlier studies.^19,21^ Tendons were first preloaded in tension to 1N and then loaded by using a displacement rate of 5 mm/min in three steps of 8% of the initial distance between the clamps (L_0_), each followed by a relaxation time of 300 s. Using an optical microscope aligned with the beam, the midsection of the callus was determined and a line scan across the sample was acquired to determine the width of each map. SAXS maps of 3 lines across the central callus width were acquired at preload, immediately after each displacement step was reached, and then at intervals of 40 s, resulting in 9 data acquisitions per strain step (Figure 1B, Supporting Information, Figure S1). Each SAXS map acquisition was 6 s (3 × 40 points resulting in a FOV of ∼0.45 × 6 mm^2^). SAXS measurements were conducted using a beam energy of 12.4 keV, beam and step size of 150 × 150 μm^2^, and exposure time of 50 ms. The scattering patterns were recorded using a Pilatus 2 M detector^44^ at a sample to detector distance of 7.138 m, providing a *q*-range of approximately 0.02 to 1.45 nm^–1^. The SAXS detector was connected to the control unit of the loading device to record the timing of SAXS acquisitions during loading, which were triggered by a custom-written acquisition macro in the control system. The beam was focused on the detector, and a beamstop was used to block the directly transmitted beam. The beam flux was measured (∼2e11 photons/s) using a glassy carbon standard specimen.^45^ As determined in our previous study on intact, uninjured rat Achilles tendons,^19^ the results should not be significantly affected by radiation damage with this setup and measurement parameters.

**Figure 1:**
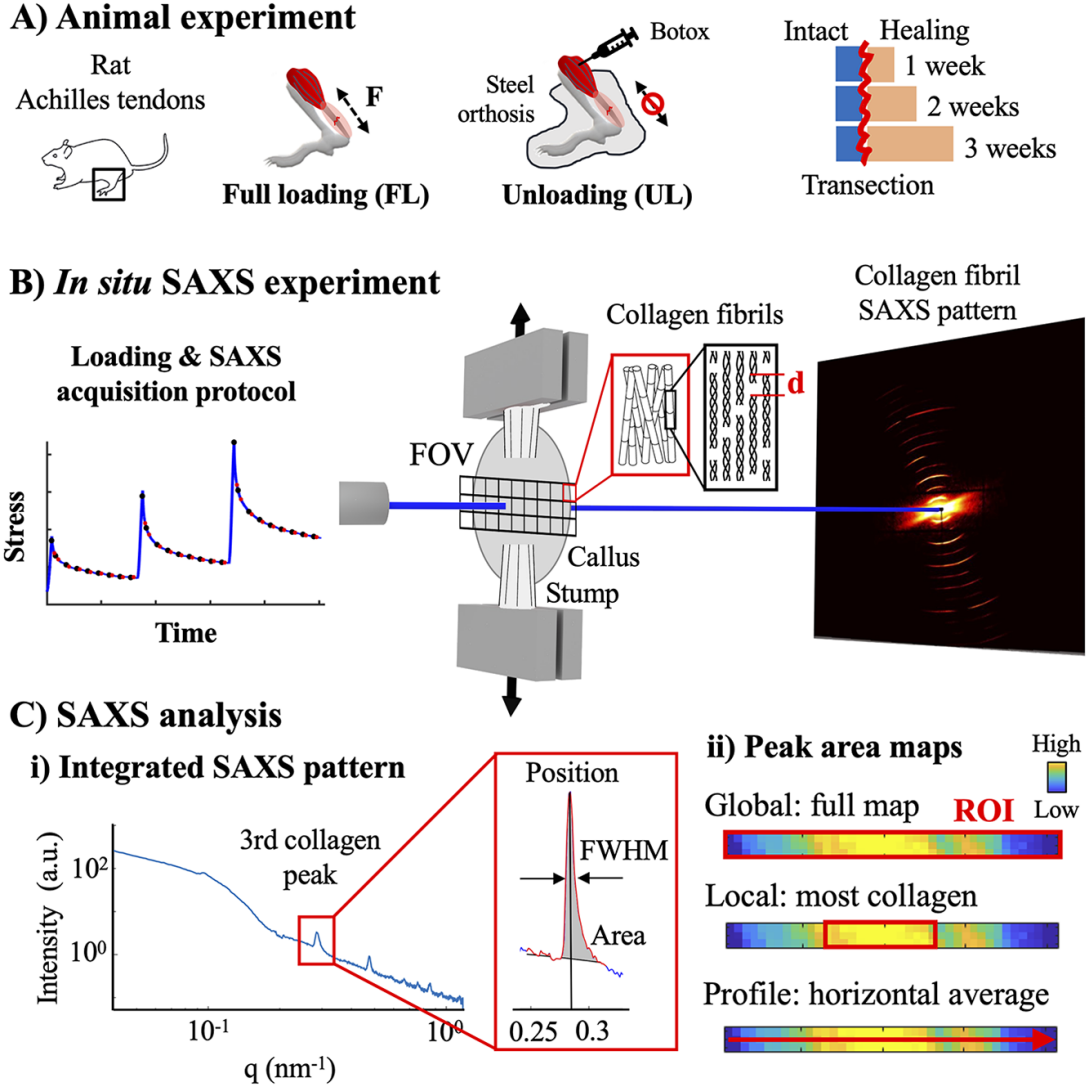
Overview of the study. (A) Achilles tendons from Sprague–Dawley rats were transected and subjected to full loading (FL) through free cage activity or complete unloading (UL) by Botox injections in combination with immobilization by steel-orthosis for 1-, 2-, and 3 weeks of healing. (B) The tendons were tested in stress relaxation, while simultaneously acquiring maps of SAXS patterns by scanning the midportion of the healing callus. (C) The 2D scattering patterns were integrated (i), and the third collagen peak was analyzed to quantify fibril *d*-spacing (peak position), amount (peak area), and strain heterogeneity (peak full-width-at-half-maximum, FWHM). The analysis was performed on two regions of interest (ii) (ROI, red boxes); the full SAXS map (global analysis, top) and a smaller region of one-third of the map including the highest collagen content (local analysis, middle), as well as horizontally across the map (profile analysis, bottom).

#### 2.2.2 Data Analysis

Analysis of the 2D scattering data was performed using in-house Matlab codes^46^ following previously described protocols.^19,21,40^ The I­(q) scattering curves used to extract collagen structural parameters were obtained in the range *q* = 0.05–1.45 nm^–1^ by radial integration over 360° of the scattering patterns using in-house Matlab codes (R2021a, MatchWorks Inc., USA).^15,46^ Fibril structural parameters such as *d*-spacing and strain heterogeneity were retrieved from the position and full-width-at-half-maximum (FWHM) of the third collagen scattering peak, respectively (Figure 1C­(i)).^15,19,46,47^ The scattering signal arises from the well-ordered repetitions of the collagen fibril *d*-spacing. Thus, the collagen scattering peak area is related to the amount as well as the gap to overlap ratio and intrafibrillar disorder of fibrils oriented in-plane (i.e., not along the direction of the beam). However, as the scattering data of the newly formed collagen was too weak to consistently provide higher order collagen peaks which would enable the analysis of the gap/overlap ratio as well as the interfibrillar disorder, the third peak area was used as a simplified indication of the presence of in-plane oriented, well-ordered fibrils within the probed volume. An increase in peak area was thus interpreted as recruitment through reorientation of out of plane fibrils and/or the fibrils becoming more well-ordered. Before quantification of the collagen parameters, the maps from all samples across all time points and groups were thresholded based on a minimum collagen peak area (i.e., fibril amount) to remove background pixels (∼10% of maximum values). The *in situ* response of all collagen parameters were evaluated at all SAXS acquisitions as a relative change in percentage compared to their respective values at preload.

The intrinsic (material-dependent) parameters stress and strain were retrieved from normalization of the extrinsic (geometry-dependent) parameters force and displacement. Tissue stress was estimated by normalizing the applied tissue force with the estimated tendon cross-sectional area (Supporting Information, Table S1). Tissue and fibril strains were obtained by normalizing the tissue displacement and changes in fibril *d*-spacing by their starting values, i.e., clamp distance (L_0_) and *d*-spacing at preload (Supporting Information, Table S1). Tissue stiffness and elastic modulus were estimated from the steepest region in the force–displacement and stress–strain curves, respectively. Tissue and fibril relaxation ratios were estimated as the relative decrease from the point of which the strain step was reached compared to the end of the relaxation period. Tissue fast relaxation times were estimated as the time at which the tissue stress had decreased to 63% of its value at the end of the relaxation period. Due to the spatial variation of collagen formation within the healing callus (Supporting Information, Figure S3), the fibril response was evaluated in three regions of interest (ROIs, Figure 1C­(ii); (1) on a global level, by averaging all data points within the SAXS map (∼0.45 × 6 mm^2^), (2) on a local level, by averaging the data points within the horizontal one-third of the SAXS map (∼0.45 × 2 mm^2^) which contained the highest collagen amount, and (3) horizontally across the callus, by averaging profiles over the height of each map. For the local analysis, the region containing the highest collagen amount was determined for each sample by computing the average within a window of one-third of the collagen peak area map at consecutive horizontal positions of the third load step and then selecting the region with the highest average value for the analysis of all parameters at all load steps. One 3 week UL tendon broke during the first step. Thus, it was excluded from both tissue and fibril mechanics. Additionally, another 3 week UL tendon included one of the stumps within the FOV of the SAXS scan. Thus, it was excluded from fibril mechanics.

### 2.3 Statistics

Mean, standard deviation (SD), and 95% confidence interval (CI) values were calculated. Due to the sample size, further statistical testing was omitted.

## 3 Results

### 3.1 Tissue Level Mechanics

At all healing time points, UL tendons showed a 40% smaller cross-sectional area and a 50% shorter stump distance compared to FL tendons (Supporting Information, Table S1). While UL tendons exhibited a larger intersample variability in both extrinsic (geometry-dependent) and intrinsic (geometry-independent) tissue parameters, they showed no clear differences compared to FL tendons in neither force or stress magnitude nor stiffness or elastic modulus (Figure 2, Supporting Information, Figure S3) despite their geometrical differences. With increasing strain steps, FL and UL tendons exhibited both increased stiffness (Figure 2(ii) and elastic modulus (Supporting Information, Figure S3­(ii)). While the stiffness in both groups increased slightly across healing time (Figure 2B­(ii), their elastic moduli remained similar (Supporting Information, Figure S3B­(ii)). The stress relaxation ratio and fast relaxation time also remained unaffected by the *in vivo* loading scheme, as well as by both strain level and healing time (Supporting Information, Figure S3B­(iii,iv)), except for after 2 weeks of healing, at which point UL tendons exhibited slightly faster relaxation times than FL tendons.

**Figure 2:**
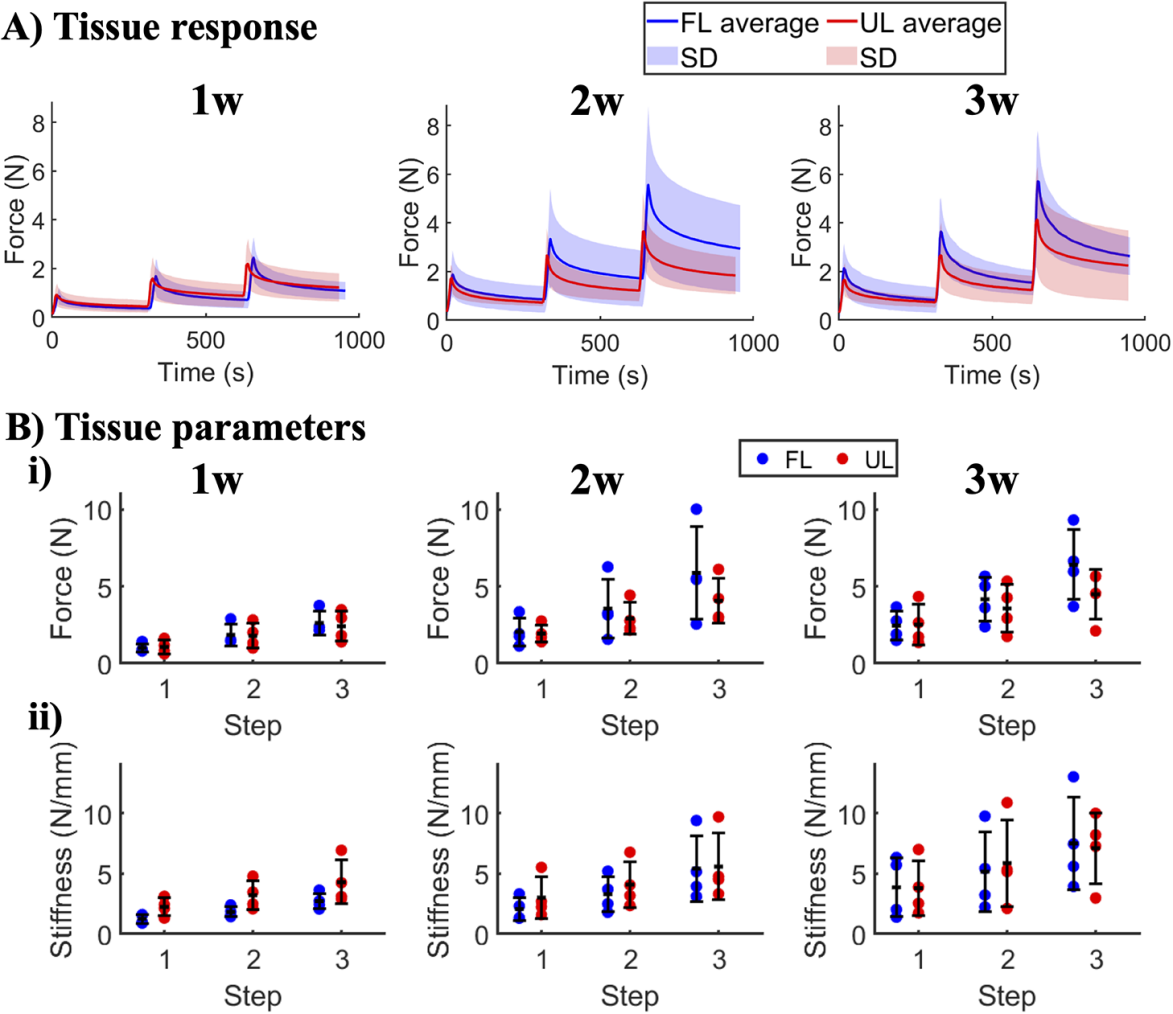
Tissue force relaxation response and mechanical properties. (A) Average tissue force-time response for the three strain steps in 1-, 2-, and 3 week healing tendons. Data are shown as mean (solid line) and standard deviation (shaded area). (B) Comparison of maximum force (i) and stiffness (ii) for each strain step. Each rat is shown as on data point for FL in blue and UL in red. Error bars represent 95% confidence interval.

### 3.2 Fibril Level Mechanics

During the first and sometimes second strain steps, UL tendons reached fibril strain levels similar to those of FL tendons (Figure 3A). However, as the strain steps increased, the fibril strain response in UL tendons remained lower than in FL tendons. This difference between the groups increased with healing time and was more prominent in the region of highest collagen amount compared to the full map (Figure 3A­(i,ii)).

**Figure 3:**
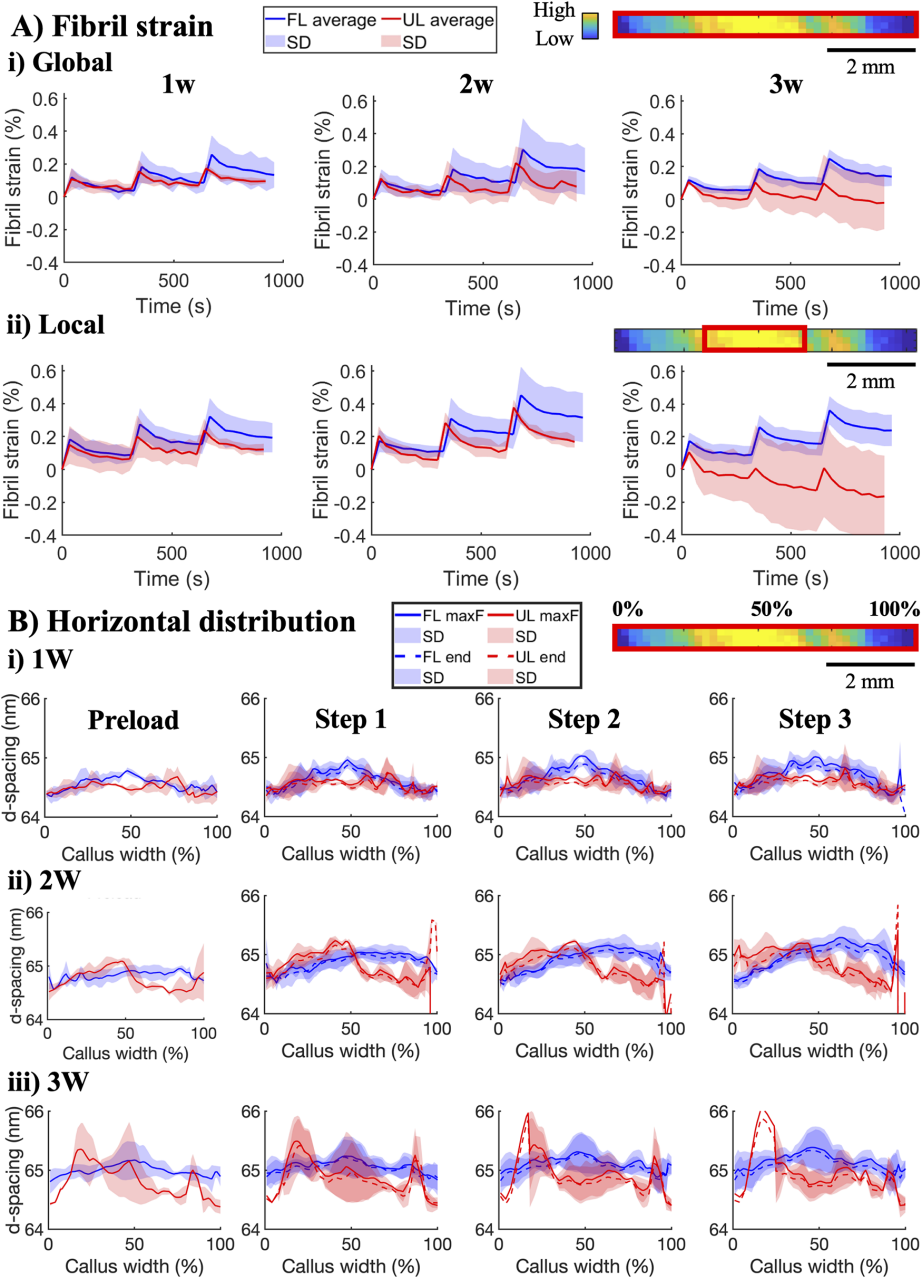
Fibril strain response to *in situ* stress relaxation. (A) Average fibril strain response on the global level of the entire FOV (i) and on the local level from the region with highest collagen content (ii) for the three strain steps in 1-, 2-, and 3 week healing tendons. Data are shown as mean (solid line) and standard deviation (shaded area). (B) Average horizontal profiles of fibril *d*-spacing across the healing callus at preload, maximum (solid line), and end of each strain step (dashed line).

Initially, both UL and FL tendons had a similar distribution of fibril *d*-spacing horizontally across the callus (Figure 3B­(i)) as well as average *d*-spacing within the full map (Supporting Information, Figure S4A­(i)). However, the *d*-spacing of UL tendons within the region of highest collagen content was initially shorter than that in FL tendons (Supporting Information, Figure S4A­(ii)). As healing progressed, the *d*-spacing of FL tendons increased homogeneously horizontally across the callus (Figure 3B), as well as locally within the region with the highest collagen amount and on average within the full map (Supporting Information, Figure S4A). On the other hand, the *d*-spacing of UL tendons exhibited more horizontal spatial variation across their calluses, typically with a higher *d*-spacing toward one side (Figure 3B­(ii,iii)). While this spatial variation in collagen maturation resulted in their average *d*-spacing of the full map remaining low compared to FL tendons (Supporting Information, Figure S4B­(i)), the *d*-spacing within the region with the highest collagen amount ultimately reached similar values as FL tendons after 3 weeks of healing (Supporting Information, Figure S4B­(ii)). Additionally, after 3 weeks of healing, UL tendons also exhibited localized regions of even higher *d*-spacing than FL tendons (Figure 3B­(iii)). No difference in fibril strain relaxation ratio was found between the FL and UL tendons (Supporting Information, Figure S4B).

Recruitment of fibrils in UL tendons remained generally low with increased tissue strain compared to FL tendons, which exhibited a clear relative increase in their collagen peak area (i.e., detected fibril amount) with increased tissue strain at all healing time points (Figure 4A). Additionally, while FL tendons exhibited an inverse relaxation in detected fibril amount during tissue relaxation, UL tendons did not exhibit (Figure 4(A,B), dashed vs solid lines).

**Figure 4:**
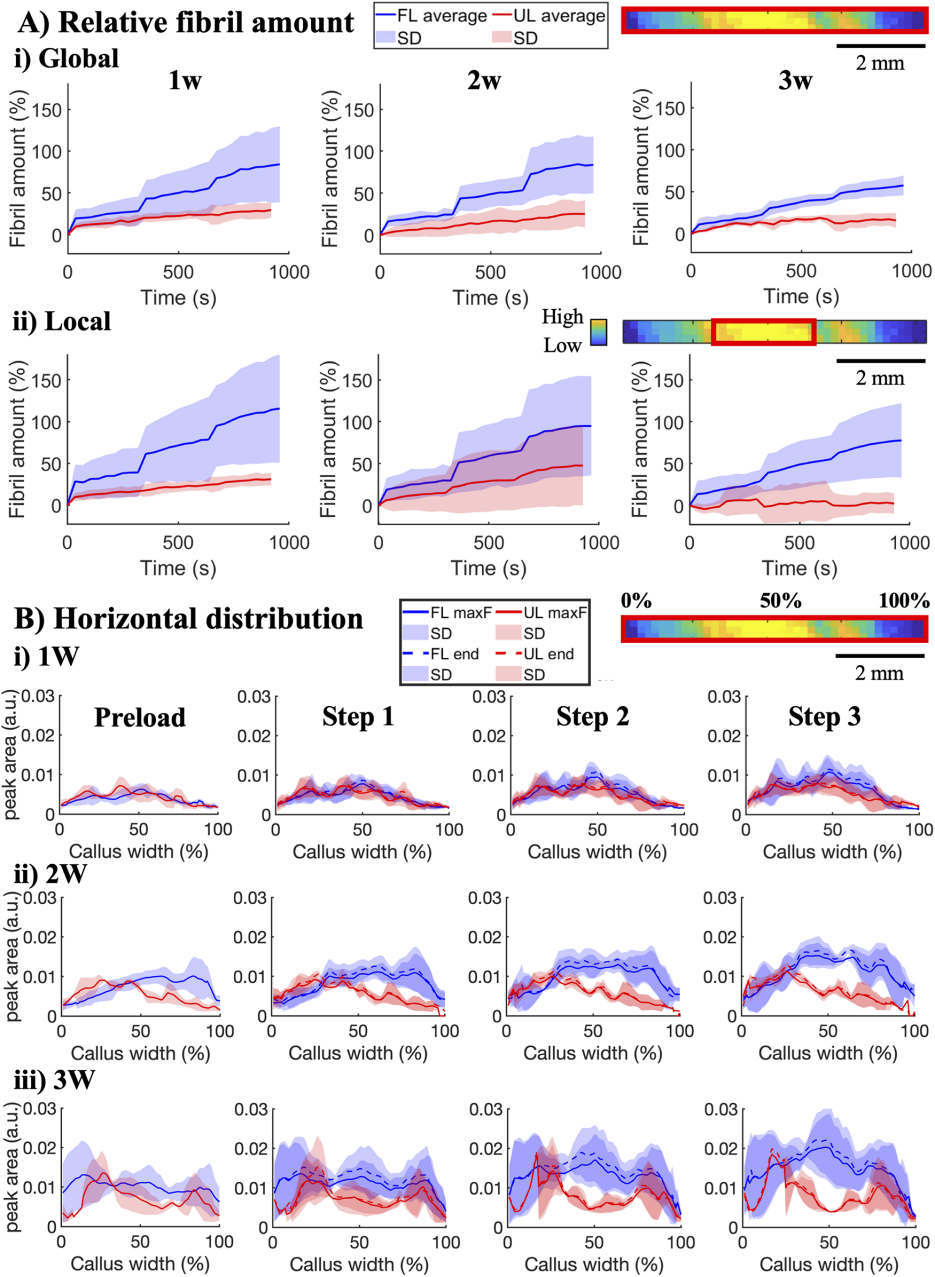
Response in detected fibril amount to *in situ* stress relaxation. (A) Average response in relative collagen peak area (i.e., detected fibril amount) on the global level of the entire FOV (i) and on the local level from the region with highest collagen content (ii) for the three strain steps in 1-, 2-, and 3 week healing tendons. Data are shown as mean (solid line) and standard deviation (shaded area). (B) Average horizontal profiles across the healing callus at preload, maximum (solid line), and end of each strain step (dashed line).

Despite their difference in relative increase with tissue strain, initially after 1 week of healing, the horizontal distribution and average detected fibril amount were similar in both UL and FL tendons (Figure 4B­(i), Supporting Information, Figure S5A). However, as healing progressed, the horizontal distribution of detected fibrils became increasingly localized in UL tendons, with the detected fibril amount increasing only on either one or both sides (external edges of the callus) instead of homogeneously across the callus as in FL tendons (Figure 4B­(ii,iii). The average detected fibril amount within both the full map as well as the region of highest collagen amount remained low in UL tendons, while it increased in FL tendons as healing progressed (Supporting Information, Figure S5A).

While the fibril strain response of FL tendons remained relatively homogeneous following the increased tissue strain steps, UL tendons exhibited a relatively more heterogeneous strain response at 1 and 2 weeks, including relaxation of the fibril strain heterogeneity (distribution of fibril *d*-spacing within the probed volume) simultaneously as tissue relaxation (Figure 5A). Despite their initial difference in their relative response to tissue strain after 1 week of healing, their horizontal distribution across the callus and average fibril strain heterogeneity were similar in both UL and FL tendons (Figure 5B­(i); Supporting Information, Figure S5B). However, as healing progressed, the absolute fibril strain heterogeneity reached during each strain step was generally lower in UL compared to FL tendons and more spatially varied across the callus (Figure 5B, Supporting Information, Figure S5B). Despite this, after 3 weeks of healing, the average relative response in fibril strain heterogeneity of UL tendons was similarly homogeneous to FL tendons (Figure 5A).

**Figure 5:**
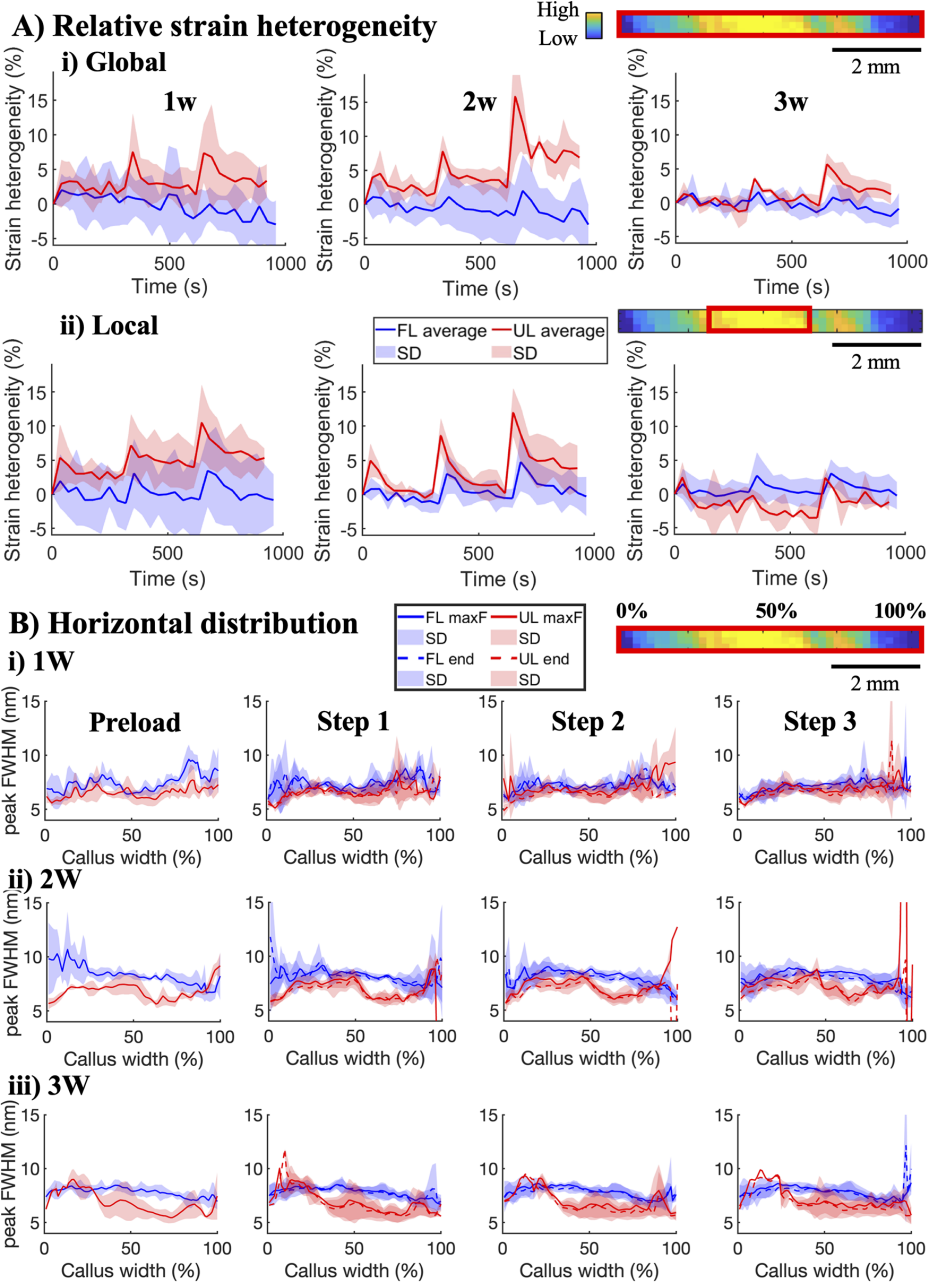
Response in strain heterogeneity to *in situ* stress relaxation. (A) Average response in fibril strain heterogeneity on the global level of the entire FOV (i) and on the local level from the region with highest collagen content (ii) for the three strain steps in 1-, 2-, and 3 week healing tendons. Data are shown as mean (solid line) and standard deviation (shaded area). (B) Average horizontal profiles of fibril *d*-spacing across the healing callus at preload, maximum (solid line), and end of each strain step (dashed line).

## 4 Discussion

This study presents the first *in situ* investigation of the spatial variations in the nanoscale mechanical response of the newly formed collagen fibrils across the early healing callus in rat Achilles tendons, with a specific focus on the effects of reduced *in vivo* loading. Except for a few recent papers on cartilage^48^ and keloid scars,^49^ to the authors knowledge, previous *in situ* SAXS investigations into the mechanical response of collagen fibrils have been limited to single spots,^20,28–35^ thus neglecting spatial variations. Here, we show that spatial variation and heterogeneity within the tissue are important in healing Achilles tendons. Our study only mapped the midsection of the healing callus, yet it still allowed us to capture the spatially heterogeneous nature of collagen formation under different *in vivo* load levels. This study provides valuable insight into the nanoscale alterations due to *in vivo* unloading during Achilles tendon healing, which were characterized by decreased fibril extensibility and recruitment, as well as higher strain heterogeneity and more extreme spatial variations. These nanoscale alterations in organization and mechanical response, could potentially be compensated for by differences in size and composition of the healing tendon on the larger length scales,^40^ to ultimately result in tendons with similar tissue response at the probed load magnitudes.

Previous studies investigating the tissue scale mechanical response of early healing Achilles tendons have indicated that unloading results in weaker tendons with impaired viscoelastic properties.^4,13–15,50^ However, in the current study, no differences in step force, stress, stiffness, elastic modulus, fast relaxation time, or relaxation ratio were observed between UL and FL tendons. This could potentially be attributed to the fact that the tissue strain steps performed in this study only reached approximately 10% of the reported failure force for 1–2 weeks healing FL tendons, but approximately 50% of the failure force for UL tendons.^13–15,50^ Thus, the similarities in tissue response observed in this study could be due to the different relative load regimes reached between the groups. However, with the current setup, accounting for both the difference in behavior between loaded and unloaded tendons as well as the progression of mechanical behavior across time is highly challenging, and thus, the strain steps were ultimately chosen to be held constant for experimental consistency. In addition to this, the small sample sizes preclude formal statistical comparison, and these findings should therefore be interpreted as a hypothesis rather than as conclusive. Furthermore, the observed increase in both stiffness and elastic modulus with increasing strain steps in both groups indicates that the tendons have not yet entered their elastic regimes. Thus, as opposed to previous studies which typically investigated load to failure, cyclic loading or creep at larger load magnitudes,^13–15,50^ only the first part of the load to failure curve was probed in this study. Additionally, we have previously observed similar indications of unaffected tendon stiffness after 2 and 4 weeks of early healing using the same animal model,^47^ and furthermore, similar observations were made for intact, uninjured rat tendons after unloading using the same experimental model.^21^ Surprisingly, while the stiffness showed a slight increase with healing time in both groups, their elastic moduli remained similar. This indicates that while more material has been added, thus increasing the geometry-dependent extrinsic properties, the tissue has not yet undergone much remodeling. This is in line with observations in patients, where the elastic modulus does not change until after the second half of the first year of healing,^51^ as well as the histological results from our previous study of the same animal model,^8^ which indicate that the tendons are primarily still in the proliferation phase. In the current study, there were still some minor differences observed in the intrinsic tissue parameters (Supporting Information, Figure S3B­(i,ii)), as some of the UL tendons exhibited an increased stress and elastic modulus compared with FL tendons. This indicates that the overall geometry and composition of the UL tendons to some degree might still compensate for the material differences observed at the nanoscale. Such a compensation is well in line with our recent histological study in the same animal model,^8^ where we found that UL tendons have a higher local staining intensity of extracellular matrix proteins such as elastin, collagen type I and III, but only the amount of collagen type III remained substantially different when normalized by the size of the healing callus. While there is no clear evidence that the *d*-spacing changes between the two collagen types, they often form hybrid fibrils. Some of these type III/I hybrid fibrils have been observed to exhibit a helicoidal supramolecular structure, more commonly found in the fibrils of tissues such as skin.^52^ Helicoidal fibrils tend to exhibit a shorter *d*-spacing of ∼65 nm due to their longer-range helical twist and larger molecular tilt (9–18° compared to 5° of fibrils typical in intact tendons). Other factors which have been shown to influence the *d*-spacing apart from changes in helical twist is the presence of intra- and intermolecular cross-links,^53^ which play an important role for the maturation of fibrils and the restoration of collagen type I tensile strength during tissue regeneration.^54^ While SAXS cannot distinguish between different collagen types nor the details of their supramolecular organization, one can hypothesize that the initially shorter *d*-spacing observed in the current study originates from the mixed collagen type III/I matrix, and that the increase in *d*-spacing with healing time marks the gradual shift into a matrix more dominated by collagen type I. The initially lower and delayed increase in *d*-spacing following unloading would thus be well in line with our observed histological results.^8^

Unloading altered and seemingly delayed collagen formation, as also observed in our previous studies^15,40^ and by others using another rat model.^55^ In line with this and other studies investigating early healing rat Achilles tendons,^4,8,13,14,40^ unloading resulted in a smaller cross-sectional area (∼40%) and shorter gap distance between the stumps (∼50%) compared to FL tendons. This difference was also reflected by a shorter total tendon length and thus resulted in an ∼50% reduction in clamp distance. Similarly to previous *ex situ* mechanical studies of healing rat Achilles tendons,^13^ −^14,15^ ,^50^ the *in situ* loading of this study was performed with a fixed displacement rate. As a result, the UL tendons were thus subjected to approximately two times faster strain rates compared to FL tendons (Supporting Information, Table S1). As tendons are strain rate dependent, this could possibly have influenced their mechanical response. However, our recent study^36^ and others^56^ have shown that at least intact, uninjured tendons only exhibit strain-rate dependent differences when the magnitude changes ∼10 times.

Our results show that collagen fibrils in both FL and UL tendons deform similarly in response to load at low tissue strain levels. At higher strain levels, the fibrils in UL tendons deformed less than those in FL tendons, exhibiting a seemingly lower capacity for fibril extension at the same tissue strain. This could be related to impaired fibril recruitment or reduced ability of the fibrils to bear load as a consequence of delayed or disrupted healing. The reduced fibril deformation in response to unloading was also observed in our previous study on intact, uninjured rat Achilles tendons.^21^ In fact, in our previous study, intact UL tendons only reached maximum fibril strains of 0.16–0.35%, compared to the 0.6–0.8% strain of intact FL tendons. Interestingly, in the current study, the healing UL tendons reached strains similar to those of the intact UL tendons of the previous study, whereas the healing FL tendons only reached approximately half of the strain of the intact FL tendons. This difference in fibril extension capacity could indicate that the higher strain levels investigated here are, in fact, reaching the elastic regime of the load curve in UL tendons, while the FL tendons are still within their toe region. In line with this, the recruited amount of collagen fibrils remained low in UL tendons at all healing time points (average of 2–45% increase in fibril amount by the third strain step), as opposed to FL tendons, which exhibited increased fibril recruitment with increased strain (average of 60–115% increase). These differences in fibril properties could be related to higher risk of reinjuries during higher loading scenarios. The fibril strain heterogeneity in UL tendons also became relatively larger with increased applied tissue strain compared to that in FL tendons, but the local strain heterogeneity reached during each strain step was smaller. Interestingly, however, both groups exhibited positive correlation between *d*-spacing and fibril amount, albeit delayed with healing time in the UL tendons (Supporting Information, Figure S6). Additionally, at 3 weeks of healing, stronger correlations were observed in UL tendons between *d*-spacing and strain heterogeneity (Supporting Information, Figure S7). This further indicates a delay in the maturation of the UL collagen network, but that the deposited material is not substantially altered. Instead, a more probable reason behind the altered extensibility, recruitment and strain heterogeneity, could be the high spatial variation in collagen amount across the callus. UL tendons exhibited increasingly larger spatial variations as healing progressed, with fibril recruitment seemingly more substantial at the periphery rather than in the midportion of the callus after 3 weeks of unloading, as opposed to FL tendons which developed and seemingly strained homogeneously across the callus. Thus, instead of having an ensemble of fibrils carrying the applied load, there might only be a few fibrils in localized clusters contributing to the load. Using phase-contrast microtomography and SAXS tensor tomography, which provide 3D structural insights into collagen fibers and fibrils, respectively, we observed spatial variations in tissue distribution and maturation correlated to unloading during early Achilles tendon healing. Moreover, these techniques also revealed increased disorganization and reduced longitudinal alignment of both fibers and fibrils, emphasizing the need for spatially resolved imaging over highly localized approaches.^40^ This is in line with effects seen on organization and mechanical response due to unloading of intact, uninjured Achilles tendons.^21^ If the fibers and fibrils are too misaligned, with, e.g., fibrils oriented away from the load direction, the tissues’ ability to recruit fibrils toward the optimal direction becomes impaired. If in addition to this, the fibers and fibrils are more entangled, it will also hinder them from extending to their full capabilities or sliding relative to each other. With just a few fibrils stretching, the relative difference between them and other unstretched fibrils is most likely larger than if all fibrils contributed to sharing the load. Furthermore, the increased load on fewer fibrils might initiate early damage or hinder their relative sliding capabilities. Thus, their disorganization together with the altered formation and maturation is most likely the reason behind the weaker fibril mechanical response of UL tendons and indicates that full loading during early healing results in a tissue with a more effective load response based on fibril recruitment and a homogeneous distribution of loads.

UL tendons exhibited a lower fibril amount, *d*-spacing, and strain heterogeneity compared to FL tendons, but in general, these alterations were more prominent when analyzing the full FOV compared to the region with the highest collagen amount. Additionally, while the extent of fibril recruitment was generally higher within the region with the highest collagen amount, the increased recruitment response of FL tendons was clearer when analyzing the full FOV. This can be explained by the larger spatial variations in collagen formation and maturity observed in UL tendons here as well as in our previous studies,^15,40^ where both reduced loading and unloading were shown to result in delayed callus maturation, disorganization and a larger spatial variation in collagen formation, with localized regions containing high levels of collagen and other regions containing more adipose tissue. Additionally, both these and the current study indicate that unloading promote collagen formation preferentially toward the peripheral regions of the callus and that in these localized regions they might reach fibril properties similar to those of FL tendons. This study shows that after 2 weeks of healing, the tendon nanoscale structure and mechanical response have been altered due to unloading, indicating that the loading regime begins to have a larger impact on tissue repair. At this stage, the fully loaded animals are loading their tendons in a manner similar to uninjured tendons, whereas during the first week they did not fully load the tendon due to a visually observed altered gait. This progression likely contributes to an even greater difference in the *in situ* response to loading between the two groups. Similar results have been observed by others through combined histological and molecular analysis, where after 2 weeks of healing the mobilized and immobilized groups started diverging in terms of tissue repair.^55^ It has also been shown that immobilization leads to a lower inflammatory response in the early stages of healing.^57^ The inflammatory response is propagated by M1 type macrophages, while M2 type macrophages are responsible for type III collagen production.^58^ It was shown previously that loading delays the shift from M1 to M2 type macrophages in rat Achilles tendons,^11^ which could explain the similarities that we see between UL and FL tendons in this study after 1 week of healing, as well as the shift into a larger heterogeneity in *d*-spacing as well as spatial fibril variations in amount and maturity in UL tendons after 3 weeks.

### 4.1 Limitations

As the allocated time provided at synchrotrons is highly limited, only a low number of samples is generally possible in this type of studies. Thus, there is a trade-off between having enough samples within a data set and enough time points, or experimental groups. In this study, we focused on including several time points, as previous studies have indicated seemingly important changes occurring during this narrow healing time frame. However, it came at the cost of a reduced number of samples per time point. Worth pointing out is that the total number of samples in this study (*n* = 24) is generally high for an *in situ* synchrotron study compared to literature.^20^ ,^30^ ,^31^ However, with the development of faster and more flexible detectors such as the newest addition of Eiger 9 M at the cSAXS beamline, the overhead time between scan lines can be reduced, and thus measurements of larger sample sets will be feasible.

The shorter gap distance in UL tendons most likely entailed SAXS FOVs being closer to the stumps compared with FL tendons, making it more difficult to determine the exact middle position between the stumps. To aid in alignment, an optical microscope was utilized to aim for the midsection; however, due to complex clamping the exact middle is difficult to determine. Additionally, as the local tissue deformation most likely concentrates at the region between the stumps, whose length varies between sample groups, normalizing the displacement using the clamp distance may result in an underestimation of the true tissue strain. However, independently of strain rate estimated from L_0_ or gap distance, the difference in strain rate between UL and FL samples was consistently ∼50% (Supporting Information, Table S1). To determine the effect of the strain rate as well as scan placement, further studies mapping the whole healing tendon need to be conducted. This would, however, significantly increase the time required per sample and result in a smaller sample size in the study, as well as higher radiation dose on each sample. Another emerging option would be to complement these studies with other full-field imaging techniques such as phase-contrast tomography, which we recently combined with *in situ* loading of intact Achilles tendons,^41^ as well as cartilage and meniscus.^59^

Lastly, this study assessed only female rats, which warrants further investigation. Previous research has demonstrated sex-based differences in tendon healing and recovery,^60^ suggesting that outcomes may differ in male animals. Consequently, future studies should include both sexes to fully characterize these effects.

## 5 Conclusions

This study provides new insight into the spatially specific response of the newly formed collagen nanostructure during early Achilles tendon healing and how this is influenced by mechanical loading. Using synchrotron-based SAXS mapping simultaneously with *in situ* loading in stress relaxation steps, we were able to capture the heterogeneous nature of collagen formation and load distribution within the healing callus. Our results show that *in vivo* unloading during early healing initially results in a similar collagen fibril structure and spatial distribution. Then as the healing time progressed, unloaded tendons exhibited more spatially specific variations. Unloading altered the nanoscale mechanical response after healing for 1 week of healing. Full loading seems to ensure a more homogeneous fibril organization and mechanical response, primarily mediated by efficient fibril recruitment and extension. On the other hand, unloading seems to impair the tissue distribution and nanoscale fibril response to load, characterized by higher spatial variations and substantially less fibril recruitment and capacity for elongation. The impaired fibril mechanical response could be due to the unloaded tendons exhibiting delayed as well as more disorganized formation and maturation, with fewer fibrils contributing to carrying the applied tissue load. Thus, the findings of this study contribute to a better understanding of the effects of *in vivo* loading on the healing outcome of the Achilles tendon.

## Supplementary Material



## Notes

Additional experimental details, including representation of SAXS acquisition protocol and global vs local map analysis, as well as supporting tissue and fibril level step results and correlation plots (PDF)

## #

I.S.B: Aix-Marseille Université, CNRS, Centrale Med, Institut Fresnel, FR-13013 Marseille, France
