# Supplementary material for: In Situ Characterization Reveals an Impaired Fibril Response to Loading Following Unloading during Early Achilles Tendon Healing
Source: ACS Biomater Sci Eng. 2026 Mar 28;12(4):2189–200. doi: 10.1021/acsbiomaterials.5c01976 (PMC13080769; doi:10.1021/acsbiomaterials.5c01976)
Supplement: Supplementary file 1 [file ab5c01976_si_001.pdf]

SUPPORTING INFORMATION

In situ characterization reveals an impaired fibril response to loading following unloading during early Achilles tendon healing

Authors: Isabella Silva Barreto[1*#], Kunal Sharma[1], Maria Pierantoni[1], Md Abdul Alim[2], Ana Diaz[3], Pernilla Eliasson[2, 4, 5], Hanna Isaksson[1*]

Affiliations:

[1] Department of Biomedical Engineering, Lund University, SE-223 63 Lund, Sweden

[2] Department of Biomedical and Clinical Sciences, Linköping University, SE-581 83 Linköping, Sweden

[3] PSI Center for Photon Science, Paul Scherrer Institute, CH-5232 Villigen PSI, Switzerland

[4] Department of Orthopaedics, Sahlgrenska University Hospital, SE-413 46 Gothenburg, Sweden

[5] Department of Orthopaedics, Sahlgrenska Academy, Gothenburg University, SE-431 80 Mölndal, Sweden

***To whom correspondence should be addressed:**

Isabella Silva Barreto, email: isabella.silva_barreto@bme.lth.se

Hanna Isaksson, email: hanna.isaksson@bme.lth.se

Department of Biomedical Engineering

Lund University / LTH

Box 118, 221 00 Lund, Sweden

SUPPORTING INFORMATION

S1. Methods

Samples

Table S1. Average physiological data of the rats used in this study. A total of 24 rats were used, with n = 4 per loading group and time point. The initial weight of the whole animal was recorded before surgery and the final weight after euthanization. Diameters used to determine cross-sectional area were measured before mounting, and L_0 as well as initial d-spacing (averaged over the full maps) were measured at preload.

	***In vivo* loading**	**1w**	**2w**	**3w**
**Initial weight (g)**	FL	296 ± 24	293 ± 3	304 ± 23
	UL	291 ± 11	299 ± 33	309 ± 4
**Final weight (g)**	FL	292 ± 18	304 ± 15	318 ± 15
	UL	274 ± 12	284 ± 29	299 ± 12
**Weight difference (%)**	FL	-1.4 ± 2.2	+3.9 ± 3.9	+4.8 ± 3.8
	UL	-5.6 ± 1.5	-5.0 ± 2.1	-3.1 ± 2.8
**Cross-sectional area (mm^2)**	FL	17 ± 2.0	19 ± 4.3	20 ± 4.0
	UL	10 ± 2.4 *(-40%)*	12 ± 2.8 *(-40%)*	10 ± 1.9 *(-50%)*
**L_0 (mm)**	FL	11 ± 1.8	11 ± 2.5	10 ± 1.6
	UL	5 ± 2.0 *(-50%)*	7 ± 2.3 *(-40%)*	9 ± 2.5 *(-10%)*
**Stump distance (mm)**	FL	10 ± 0.05	10 ± 1.5	10 ± 1
	UL	5 ± 1 *(-50%)*	5 ± 0.2 *(-50%)*	5 ± 1.5 *(-50%)*
**Strain rate ($\%L_0 \cdot s^{-1}$)**	FL	0.8 ± 0.1	0.7 ± 0.1	0.9 ± 0.1
	UL	1.7 ± 0.5 *(+130%)*	1.2 ± 0.4 *(+70%)*	1.0 ± 0.2 *(+20%)*
**Strain rate (%Stump dist. $\cdot s^{-1}$)**	FL	0.8 ± 0.0	0.8 ± 0.1	0.8 ± 0.1
	UL	1.9 ± 0.7 *(+120%)*	1.7 ± 0.1 *(+110%)*	2.0 ± 0.7 *(+130%)*
**d-spacing (nm)**	FL	64.6 ± 0.06	64.8 ± 0.08	65.0 ± 0.10
	UL	64.5 ± 0.08 *(-0.15%)*	64.8 ± 0.20 (-)	64.9 ± 0.18 *(-0.15%)*

SUPPORTING INFORMATION

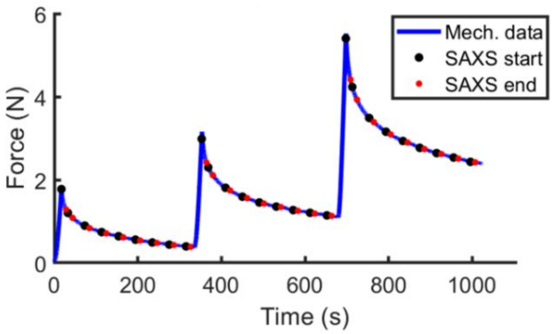

Supplementary Figure S1. SAXS acquisition protocol. Representative force relaxation curve with timing of SAXS acquisitions, indicating the start of the first SAXS line scan (black dot) and the end of the third SAXS line scan (red dot). The majority of scan time spent is due to the overhead between scan lines.

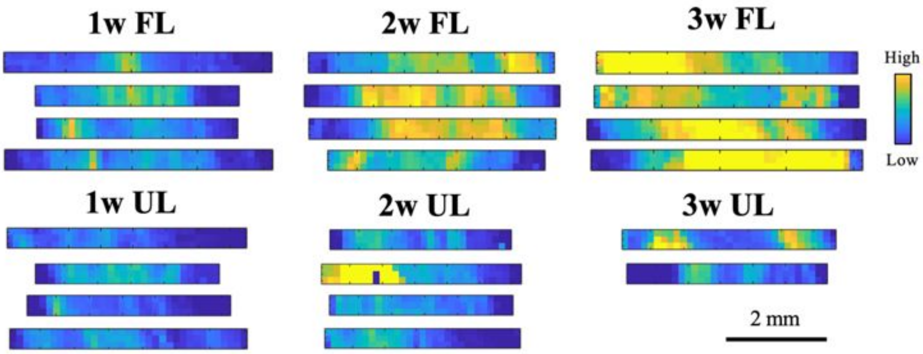

Supplementary Figure S2. Distribution of fibril amount within the healing callus of the different animals. Collagen peak area (i.e fibril amount) within the scanned regions at the point at which the 3rd strain step is reached.

S2. Results

Tissue level mechanics

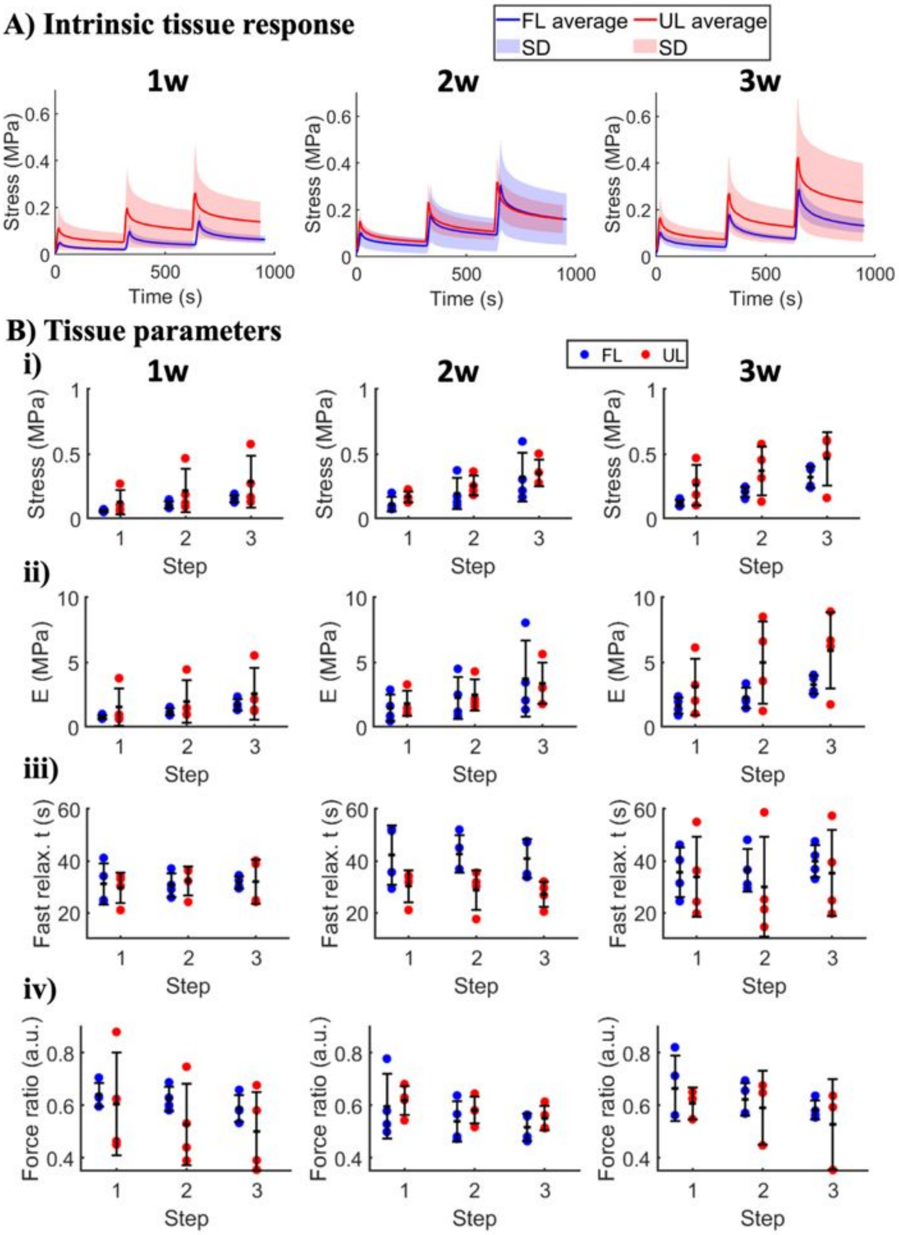

Supplementary Figure S3. Tissue mechanical response and properties. A) Average tissue stress-time response for the three strain steps. Data is shown as mean (solid line) and standard deviation (shaded area). B) Comparison of maximum stress (i), elastic modulus E (ii), fast relaxation time (iii) and relaxation ratio (iv) of each step. Each rat is shown as on data point for FL in blue and UL in red. Error bars represent 95% confidence interval.

Fibril level mechanics

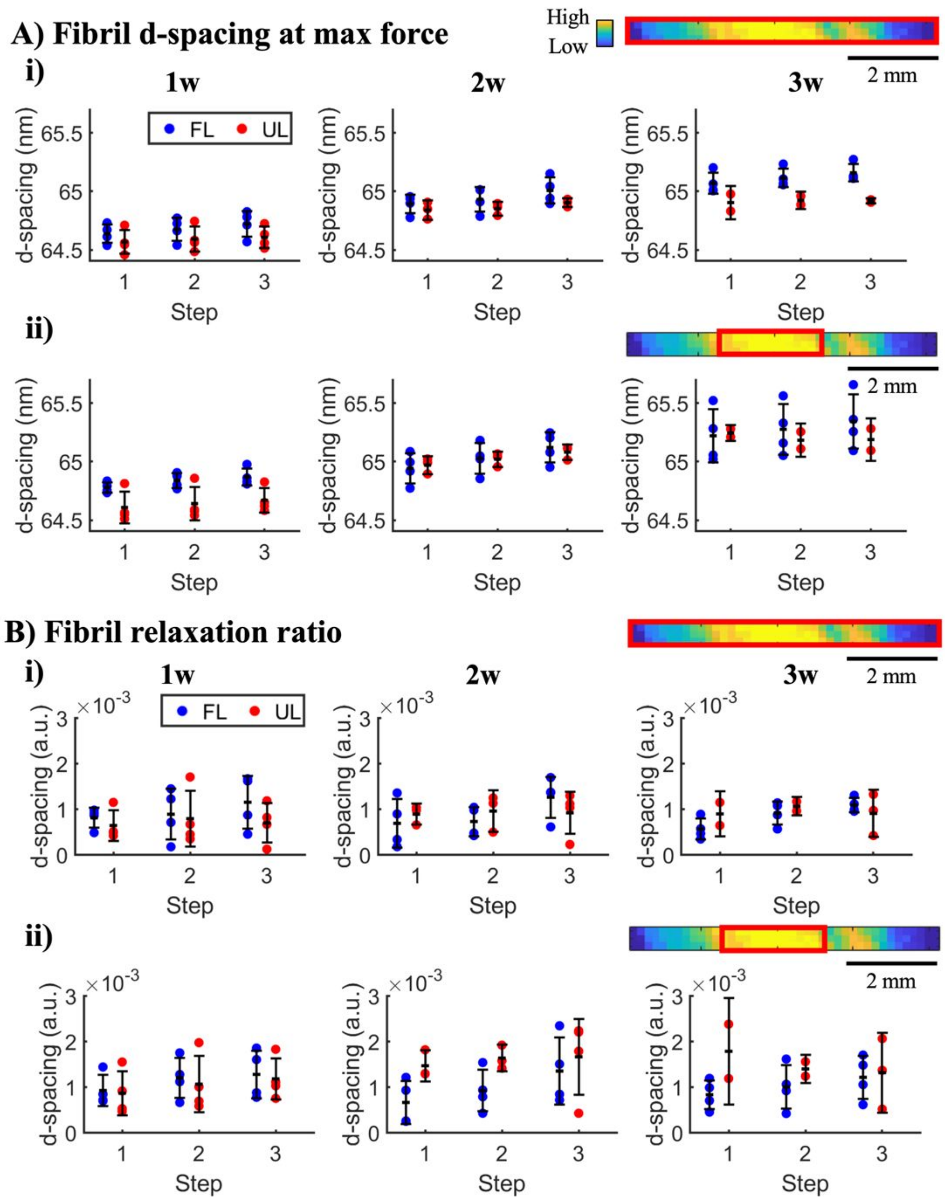

Supplementary Figure S4. Fibril d-spacing and relaxation. A) Comparison between the fibril d-spacing reached for each strain step and B) d-spacing relaxation ratios of each strain step on the global (i) and local (ii) level. Each rat is shown as on data point for FL in blue and UL in red. Error bars represent 95% confidence interval.

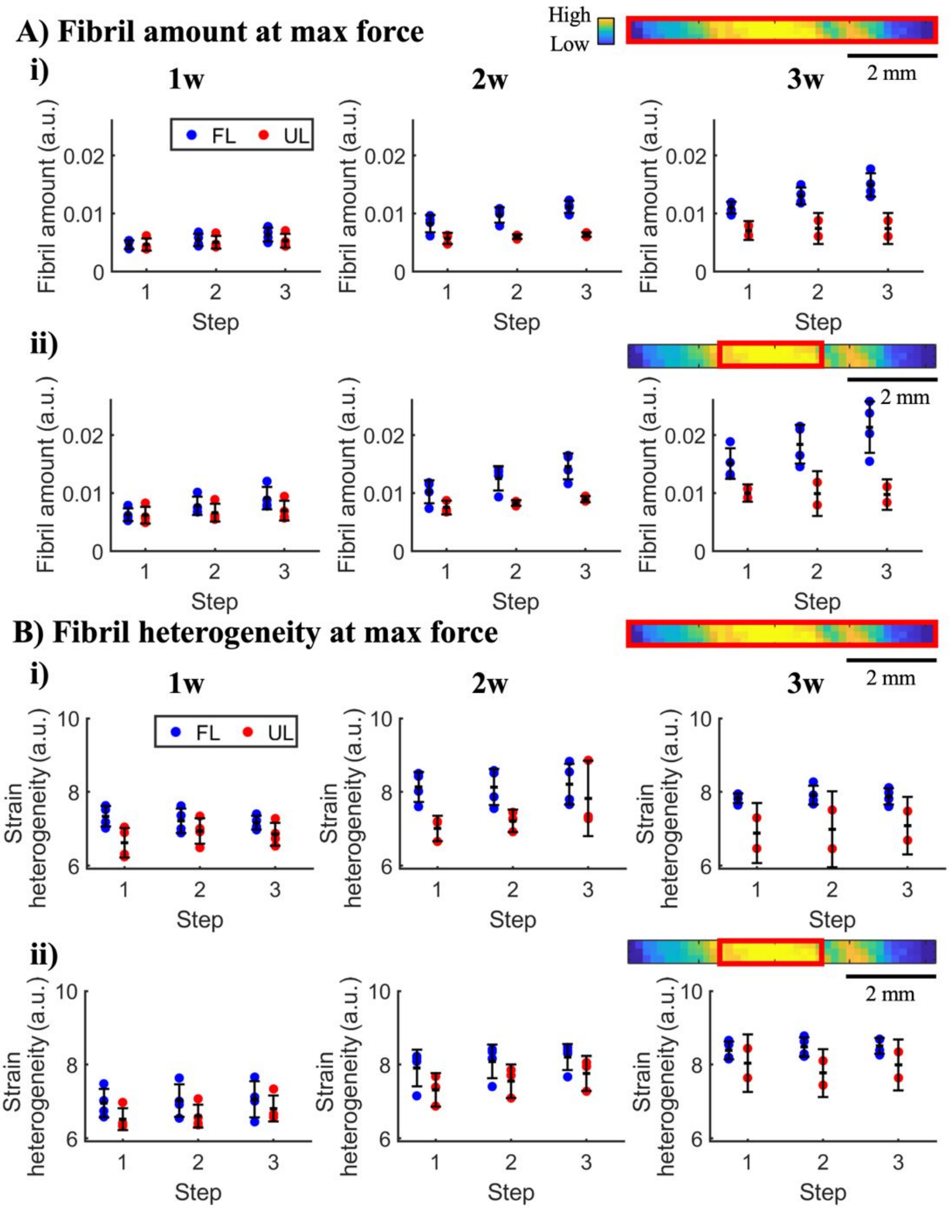

Supplementary Figure S5. Fibril amount and heterogeneity. Comparison between the A) collagen peak area (fibril amount) and B) peak FWHM (d-spacing heterogeneity) reached for each strain step on the global (i) and local (ii) level. Each rat is shown as on data point for FL in blue and UL in red. Error bars represent 95% confidence interval.

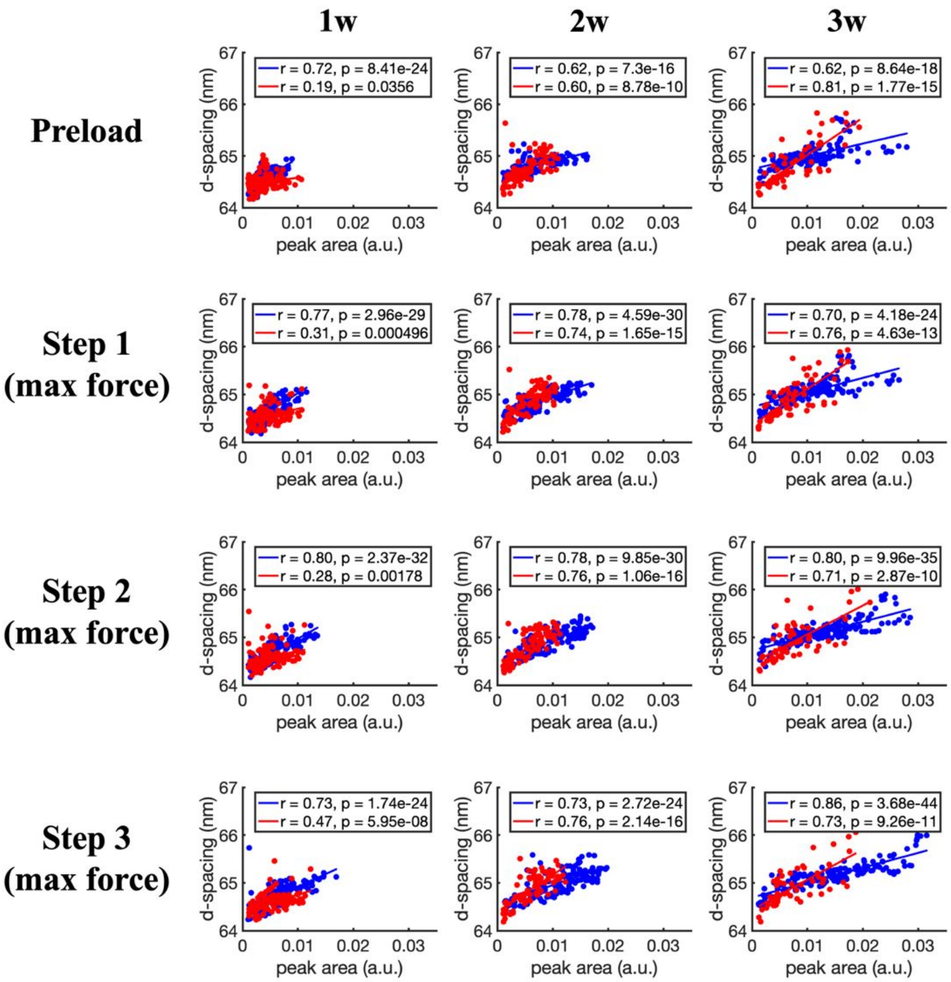

Supplementary Figure S6. Correlation between d-spacing and collagen peak area. Comparison between FL and UL callus d-spacing and collagen peak area at preload and max force at step 1, 2 and 3, including a linear fit of the data and the Pearson correlation coefficient r as well as its p-value.

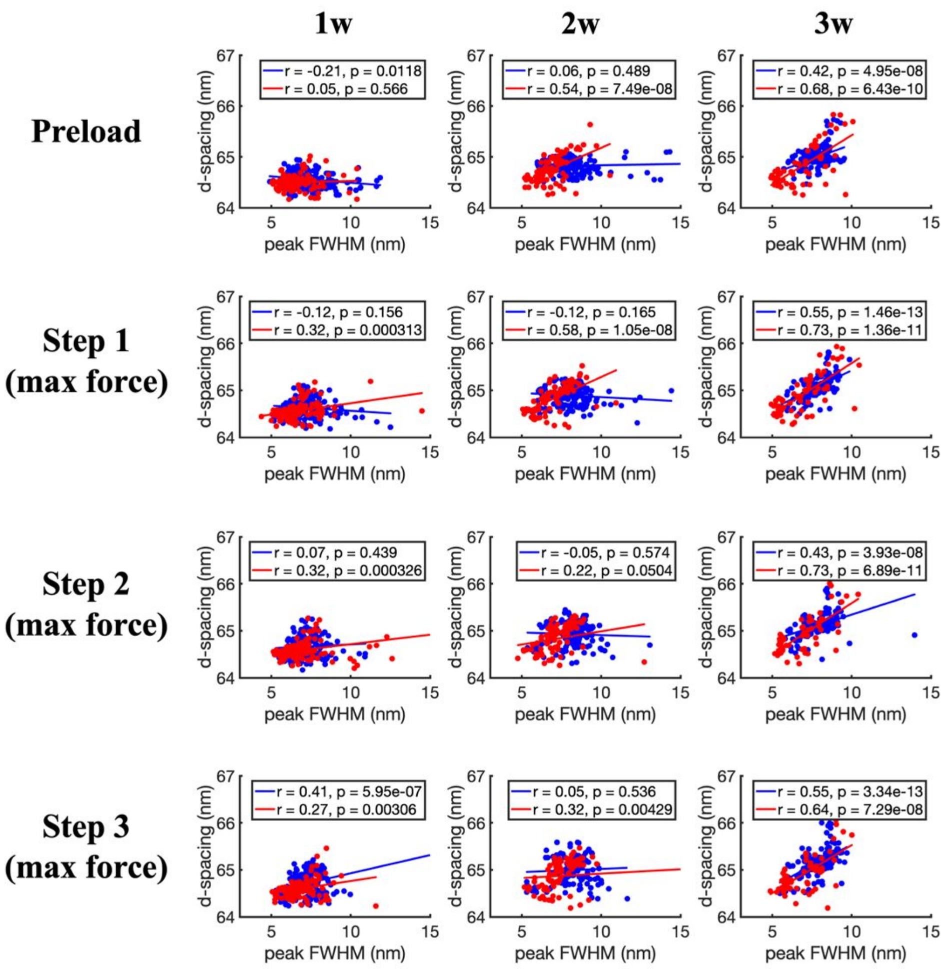

Supplementary Figure S7. Correlation between d-spacing and collagen peak FWHM. Comparison between FL and UL callus d-spacing and collagen peak FWHM at preload and max force at step 1, 2 and 3, including a linear fit of the data and the Pearson correlation coefficient r as well as its p-value.

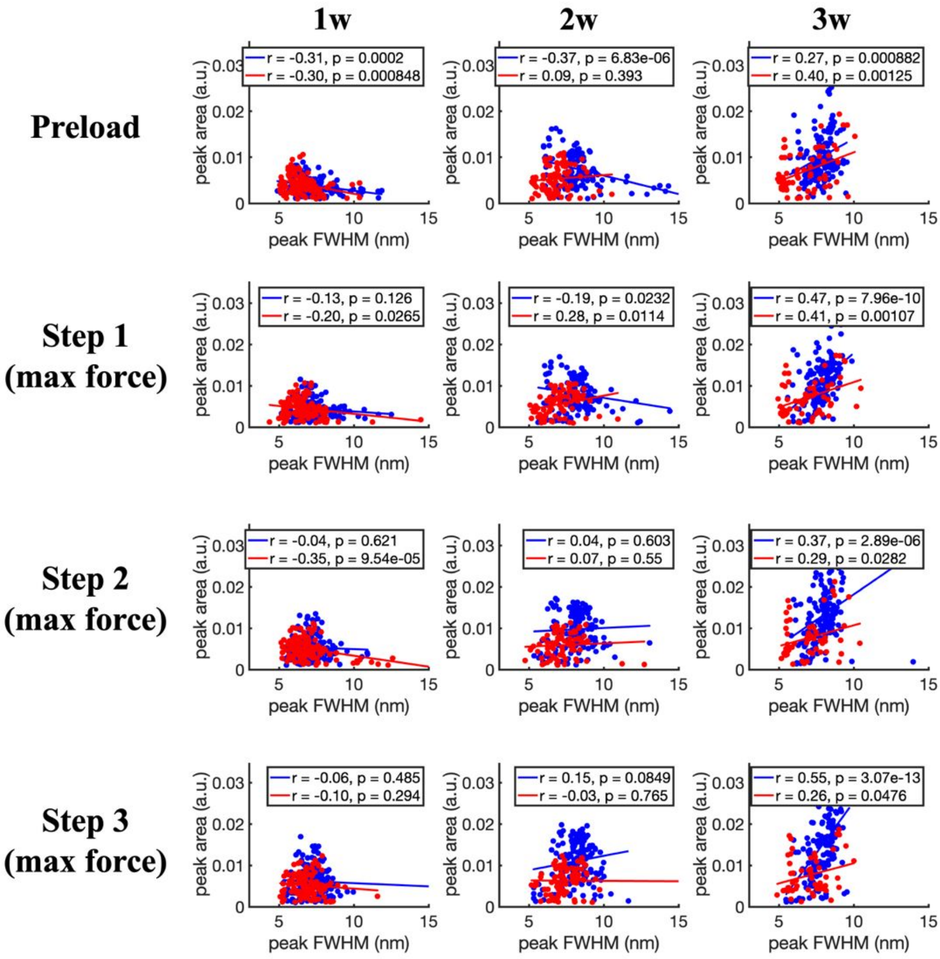

Supplementary Figure S8. Correlation between collagen peak area and FWHM. Comparison between FL and UL callus collagen peak area and FWHM at preload and max force at step 1, 2 and 3, including a linear fit of the data and the Pearson correlation coefficient r as well as its p-value.
